# Yeast adaptation to weak acids prevents futile energy expenditure

**DOI:** 10.3389/fmicb.2013.00142

**Published:** 2013-06-11

**Authors:** Azmat Ullah, Gayathri Chandrasekaran, Stanley Brul, Gertien J. Smits

**Affiliations:** Department of Molecular Biology and Microbial Food Safety, Swammerdam Institute for Life Sciences, Netherlands Institute for Systems Biology, University of AmsterdamAmsterdam, Netherlands; Food Science Department, Government College University FaisalabadFaisalabad, Pakistan

**Keywords:** weak organic acid stress, food preservatives, intracellular pH, spoilage yeast, *Saccharomyces cerevisiae*, bioenergetics, plasma membrane pumps

## Abstract

Weak organic acids (WOAs) are widely used preservatives to prevent fungal spoilage of foods and beverages. Exposure of baker's yeast *Saccharomyces cerevisiae* to WOA leads to cellular acidification and anion accumulation. Pre-adaptation of cultures reduced the rate of acidification caused by weak acid exposure, most likely as a result of changes in plasma membrane or cell wall composition. In order to adapt to sublethal concentrations of the acids and grow, yeast cells activate ATP consuming membrane transporters to remove protons and anions. We explored to what extent ATP depletion contributes to growth inhibition in sorbic or acetic acid treated cells. Therefore, we analyzed the effect of the reduction of proton and anion pumping activity on intracellular pH (pH_i_), growth, and energy status upon exposure to the hydrophilic acetic acid (HA) and the lipophilic sorbic acid (HS). ATP concentrations were dependent on the severity of the stress. Unexpectedly, we observed a stronger reduction of ATP with growth *reducing* than with growth *inhibitory* concentrations of both acids. We deduce that the not the ATP reduction caused by proton pumping, but rather the cost of sorbate anion pumping contributes to growth inhibition. A reduction of proton pumping activity may reduce ATP consumption, but the resulting decrease of pH_i_ affects growth more. ATP utilization was differentially regulated during moderate and severe stress conditions. We propose that the energy depletion alone is not the cause of growth inhibition during HA or HS stress. Rather, the cells appear to reduce ATP consumption in high stress conditions, likely to prevent futile cycling and maintain energy reserves for growth resumption in more favorable conditions. The mechanism for such decision making remains to be established.

## Introduction

Every year, large quantities of food are contaminated by microbes and become unfit for human consumption. Food spoilage microbes in large-scale food industries is prevented among other methods with weak organic acids (WOAs) such as sorbic, acetic, benzoic, and propionic acid, which are considered to be safe for human consumption. In aqueous solution, WOAs exist in pH-dependent equilibrium between uncharged acidic molecules and their charged anions. The protonated, uncharged molecule that is abundant at low pH can freely permeate the plasma membrane. Upon encountering the high pH (~7) inside the cell the acid dissociates to form a charged proton and anion which diffuse over the membrane anymore (Brul and Coote, 1999; Lambert and Stratford, 1999; Orij et al., 2009). Diffusion of weak acids into the cell could theoretically continue until equilibrium is reached between the concentration of the protonated state inside and outside the cell.

A first consequence of WOA stress is cellular acidification [reviewed in Piper et al. (2001), Orij et al., 2011]. Intracellular pH (pH_i_) affects many cellular processes, and even a slight deviation of pH_i_ already affects intracellular metabolic reactions, as it influences the ionization states of acidic and basic side chains of amino acids and thereby protein activity (Orij et al., 2011). pH_i_ is also a critical component of the total electro-chemical gradient which is responsible for the transport of molecules across membranes (Orij et al., 2011). The release of protons and subsequent cytoplasmic acidification leads to inhibition of essential metabolic functions (Krebs et al., 1983; Bracey et al., 1998), inhibition of glycolysis (Pearce et al., 2001) and therefore reduction of the cell's ability to generate ATP. In addition, the cellular activities counteracting acidification and anion accumulation consume ATP (Piper et al., 1998; Holyoak et al., 1999). In yeast intracellular acidification is partly counteracted by the activity of Pma1p, a plasma membrane H^+^-ATPase pump. *PMA1* is an essential gene that encodes the major pH_i_ regulator in baker's yeast (Serrano et al., 1986). It pumps H^+^ ions out of the cell using ATP hydrolysis at a 1:1 ratio (de Kok et al., 2012). This activity consumes about 20% of the ATP produced during normal conditions (Morsomme et al., 2000) and up to 60% during weak acid stress (Holyoak et al., 1996). Lipophilic WOA stress also induces the plasma membrane ATP-binding cassette (ABC) transporter Pdr12p (Hatzixanthis et al., 2003), which is believed to play a role in the adaptation of *Saccharomyces cerevisiae* to these weak acids by pumping out anions (Holyoak et al., 1999) at the cost of energy, either ATP or an aspect of membrane potential, possibly the proton gradient (Breeuwer et al., 1994; Henriques et al., 1997). *PDR12* was shown to be important for the adaptation of yeast cells to grow in the presence of lipophilic weak acid preservatives, and *pdr12*Δ mutants are hypersensitive to lipophilic acids at low pH (Holyoak et al., 1999; Hatzixanthis et al., 2003). The deletion mutant is however not sensitive to highly lipophilic, long-chain fatty acids alcohols and dicarboxylic acids (Hatzixanthis et al., 2003), and induction of the protein is not sufficient for growth in the presence of sorbic acid (Papadimitriou et al., 2007).

Once outside, the protons and anions exported from the cell will re-associate due to the low medium pH, and can diffuse back over the membrane. This constitutes a futile cycle that will provide a constant drain of the cell's energy reserves. Non-futile ATP consumption should lead to decreased diffusional entry of acid, and a decrease of pumping activity might lead to a reduced ATP burden (Cole and Keenan, 1986; Piper et al., 2001; Mira et al., 2010b). Indeed, several genome-wide analyses of the effects of WOA stress on yeast have revealed that the energy burden is significant, and that this might be a major cause of the growth inhibitory effect (Mollapour et al., 2004; Schüller et al., 2004; Mira et al., 2010a), and the effects of weak acids on yeast growth are observed as a linear relation between WOA concentration and maintenance energy requirement (Quintas et al., 2005). In this study we aimed to determine whether the accumulation of protons and anions in the cell, or rather the depletion of ATP caused by the export of both is the cause of the growth inhibitory effect of these acids.

## Materials and methods

### Yeast strains and plasmids

*S. cerevisiae* BY4741 (*MATa his*3Δ1 *leu*2Δ0 *met*15Δ0 *ura*3Δ0) and the derivatives *pma1-007* (*YGL007W*::kanMX4) and *pdr12*Δ (*YPL058C*::kanMX4) were used in this study (EUROSCARF, Germany). All strains were transformed with plasmid pYES-ACT-pHluorin to measure pH_i_ as described earlier (Ullah et al., 2012). All chemicals were purchased from Sigma–Aldrich (Germany), unless otherwise indicated.

### Growth conditions

Cells were grown at 30°C in a defined mineral media medium according to (Verduyn et al., 1992) using 2% glucose. Pre-inocula were grown overnight in Erlenmeyer flasks shaking at 200 rpm in the same media as above, buffered at pH 5.0 with 25 mM potassium citrate. Strains were cultivated in 500 ml batch fermenters to an initial OD_600_ of 0.1, with a steady airflow (500 ml/min) and stirring rate (600 rpm). External pH was maintained at 5.0 by titration with 0.2 M KOH using Applikon ADI 1030 Controller (Applikon, Schiedam, the Netherlands). Cultures were exposed to WOA in early exponential phase (OD_600_ ~1.0) as indicated.

### Measurement of OD_600_ and pH_i_

pH_i_ was measured as described before (Orij et al., 2009). Growth (OD_600_) and pH_i_ were monitored at regular intervals for 4 h. Culture samples from the batch fermenters were transferred to CELLSTAR black polystyrene clear bottom 96-well microtiter plates (BMG Labtechnologies, Germany). OD_600_ and pH_i_ of the cultures were measured in a FLUOstar Optima microtiter plate reader (BMG Labtechnologies, Germany) in three technical replicates.

### Determination of acidification rate

Growing cultures of *S. cerevisiae* were exposed to HA (45 mM) and HS (1 mM), or control conditions at pH 5.0 and 30°C for 4 h. Cells were harvested by centrifugation at 5000 rpm for 5 min, washed, and suspended in fresh medium (with or without glucose). Pre-exposed cultures were pulsed with WOA and pH_i_ was monitored at one-second intervals.

### Nucleotide extraction

Samples for extraction of metabolites were collected at *t* = 0, 0.5, 1, 2, 4 h after exposure of cultures to WOAs. Metabolites were extracted with boiling ethanol after quenching in methanol (Gonzalez et al., 1997). Briefly, pre-weighed cell culture samples (20 ml) were quenched using 60% (v/v) ice cold aqueous methanol (60 ml). Samples were centrifuged at 5000 g for 5 min at −20°C. Boiling ethanol 75% (v/v) was added to the pellet and incubated for 3 min at RT. After centrifugation for 5 min at −10°C the supernatant was transferred to eppendorf tubes. The liquid was evaporated using a Speed-Vac, the residue was reconstituted in 180 μl of demi water, and stored at −80°C until further analysis.

### Nucleotide quantification

Metabolite measurements were performed by fluorimetric detection of NADH/NADPH using appropriate coupling enzymes (Gonzalez et al., 1997). Emission was measured at 460 nm after excitation at 340 nm using a FLUOstar Optima microtiter plate reader (BMG Labtechnologies, Germany).

Enzymatic determination of ATP was done at 30°C in TEA buffer (66 mM, pH 7.6) containing 6.6 mM MgSO_4_ and 0.65 mM NADP^+^. ATP was consumed by hexokinase (1.9 U/ml) in reaction with glucose (0.1 mM). The reaction reached an end point after 10 min, and NADH concentrations were determined.

ADP was also determined at 30°C in the buffer containing 66 mM TEA-pH 7.6, 6.6 mM MgSO_4_, 66 mM KCl, 0.2 mM NADH, and lactate dehydrogenase (1.8 U/ml). This step eliminates pyruvate and converts it into lactate, after completion of the reaction 0.2 mM phosphoenolpyruvate and pyruvate kinase (1.8 U/ml) was added to measure the levels of ADP. The end point of this reaction was found to be 40 min after enzyme addition. The concentration of ATP and ADP was expressed in μmol/3 × 10^7^ cells/ml assuming the number of cells at OD_600_ 1 was ~3 × 10^7^ cells (Orij et al., 2009). The recovery of the ATP after extraction was 90.75% and this represents the efficiency and stability of the metabolite during this extraction procedure (Table 1).

**Table 1:** **Metabolite recovery after extraction in boiling ethanol coupled with 60% methanol quenching**.

**Sample**	**Exogenous ATP added**	**Measured ATP**	**Difference**	**Recovery**
1	0	8.40 ± 0.40	−	−
2	5	12.00 ± 0.80	3.60 ± 1.02	72 ± 20%
3	7.60	14.40 ± 0.36	6.00 ± 0.30	78 ± 4%
4	10	17.20 ± 1.18	8.80 ± 1.50	88 ± 15%

## Results

### Adaptation to weak acids increases passive defense mechanisms

**Figure 1:**
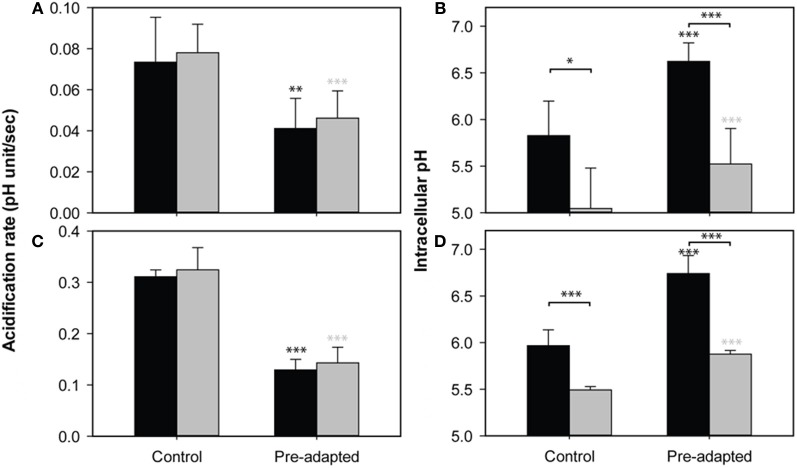
**Effect of WOA pre-exposure on acid entry**. The rate of acidification **(A,C)** and pHi reduction after 1 min **(B,D)** caused by 20 mM acetic acid **(A,B)** or 1.2 mM sorbic acid **(C,D)**, in control cultures (no pre-exposure) and culture pre-exposed to 45 mM of HA **(A,B)** or 1 mM of HS **(C,D)**. The same experiment was performed in the presence (black) and absence (gray) of glucose. Data presented are the average of three independent cultivations and error bars represent standard deviations. An asterisk indicates significance of the difference between control and pre-adapted cultures, or between conditions indicated with a marker (two-tailed *t*-test; ^*^p < 0.05; ^**^p < 0.01; ^***^p < 0.001).

*S. cerevisiae* acquires resistance to WOAs by activating the membrane H^+^-ATPase Pma1p and transporters to expel anions. Both processes cost energy. Baker's yeast does not degrade or metabolize the WOAs in the presence of glucose (Mollapour et al., 2008), so this energy dependent extrusion will be futile unless cells restrict the diffusional entry of WOA or increase the cellular capacity for absorption of the stress, for instance by increasing the concentration of buffering metabolites. Therefore, we probed the cells' ability to restrict the diffusional entry of weak acids. We have used two different WOAs, sorbic acid and acetic acid. Both acids have different toxic effect despite their identical pK_a_ values. HA inhibits growth because of its effect on cytosolic pH, whereas HS affects growth mostly by anion specific inhibitory effects (Ullah et al., 2012). We pulsed HA and HS to growing cultures of *S. cerevisiae* and estimated the initial entry by determination of the rate of intracellular acidification (Figures 1A,C). We confirmed that HS acidified cells faster than HA. Next, we pre-exposed the cultures to 45 mM of acetic acid or 1 mM sorbic acid, both of which inhibit growth by ~50%, to induce the adaptive response. After 4 h of incubation, when the pH_i_ recovery is maximal [back to ~6.5 in both cases, see Ullah et al. (2012)], cells were washed, suspended into fresh media and pulsed with 20 mM and 45 mM of HA or 1 or 1.2 mM of HS (Figure 1). We show only the results from the 20 mM HA pulse, as 45 mM in the absence of glucose resulted in pH_i_ values that were outside of the range of the calibration curve (our unpublished data). The reduction of acidification rate by the pre-exposure was, however, similar. The rate of acidification was reduced by 50%, and pre-exposure to both acids caused the cells to have a higher pH_i_ 1 min after an acid pulse. For HS, the results with 1 mM or 1.2 mM were similar (our unpublished data), but the reduction in entry rate was more apparent in case of the 1.2 mM pulse.

A reduction of the rate of acidification can be accomplished in two ways: Membranes can be altered to reduce the entry of the acids (Mira et al., 2010a), or the induction of (proton) pumping activity balancing the entry also reduces acidification (Ullah et al., 2012). To distinguish between these options, we compared the acidification of pre-exposed and control cultures of *S. cerevisiae* wild-type in the presence and absence of glucose. Removal of glucose from medium leads to the depletion of the ATP pool within minutes (Ashe et al., 2000; Thomsson et al., 2005), and thereby disables the pumping activity. Any differences in initial acidification upon WOA pulses under these circumstances should be caused by changes in influx. Although in the absence of glucose pH_i_ was reduced more strongly than in the presence of a source of energy, the *rate* of acidification was virtually identical in both situations (Figure 1). In contrast, the effect of the adaptive response to WOAs induced by pre-exposure caused a decrease in the rate of acidification, which was again identical with or without glucose. This decrease in acidification rate is therefore not dependent on ATP dependent processes, and must be caused by a decreased diffusional entry of WOAs by remodeling of the cell surface. It should be noted that while the absence of an energy source did not affect the acidification rate, the pH_i_ reached after 1 min was lower in cultures without glucose (Figures 1B,D). Therefore, while the initial acidification rate, reflecting diffusional entry, is reduced by the pre-adaptation, adapted cells could not completely overcome the diffusional entry, and still need energy for the maintenance of pH_i_ in the presence of WOA. This energy demand might result in depletion of cellular ATP and therefore in reduced growth.

### WOAs lead to ATP depletion at low dosages

**Figure 2:**
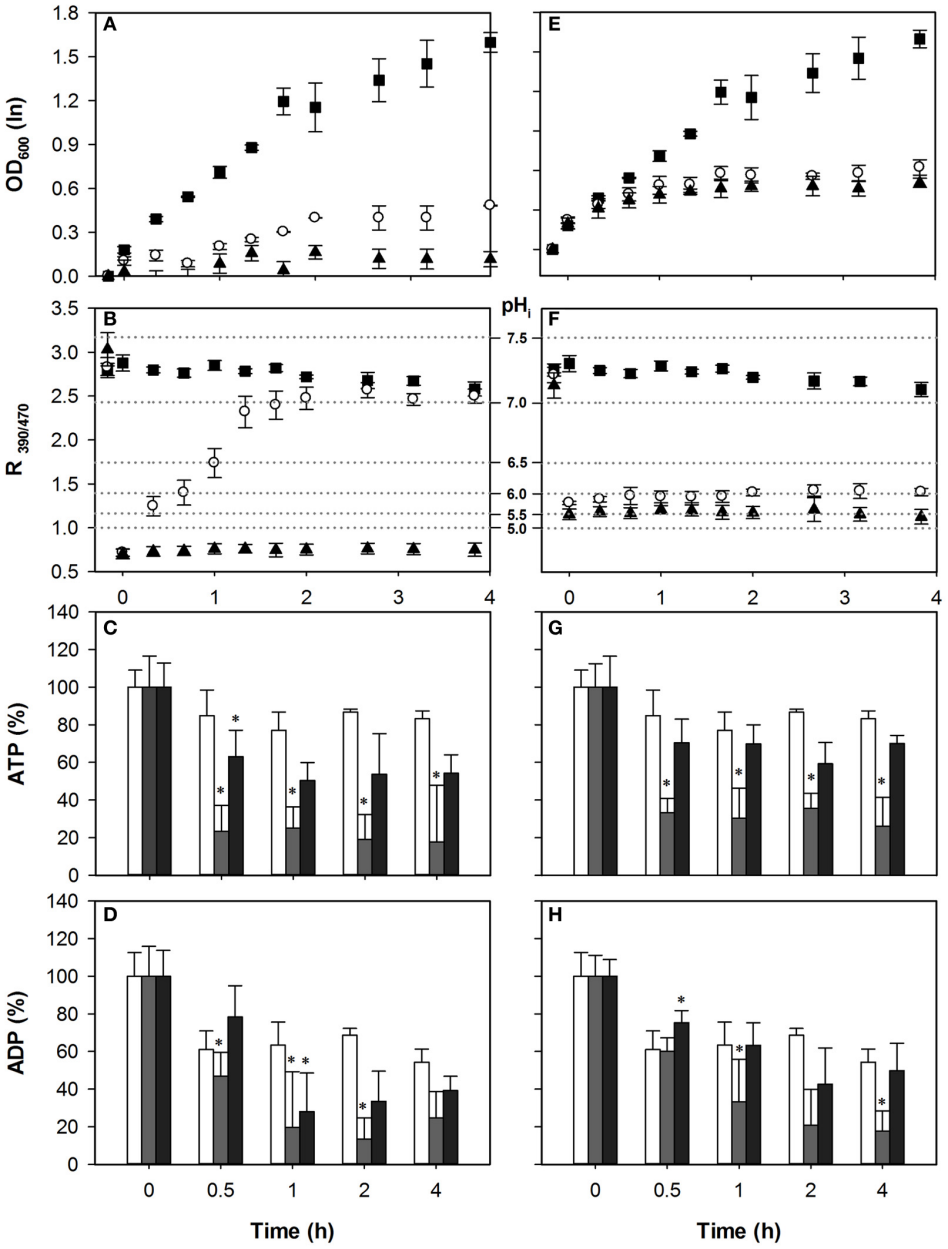
**Effect of acetic acid (left hand panels) and sorbic acid (right hand panels) on growth (A,E), intracellular pH (B,F), ATP (C,G), and ADP (D,H) levels of BY4741**. Growing cultures were exposed to 0 mM (■ and white bars), 40 mM HA or 1 mM HS (◦ and gray bars), and 70 mM HA or 2 mM HS (▲ and dark gray bars) at *t* = 0. pH_i_ data are presented as ratio of fluorescence emission upon excitation at 390 nm and 470 nm (R_390/470_) while the relevant pH_i_ values derived from the calibration curves are also indicated. Data presented are the average of three independent cultivations and error bars represent standard deviation. An asterisk indicates significance of the difference between acid exposed and control cultures at the same time point (two-tailed *t*-test; *P* < 0.05).

To test whether the ATP consumption for counteracting acid stress is a major cause of growth inhibition by weak acids we measured the intracellular concentrations of adenine nucleotides (ATP, ADP), and simultaneously monitored growth and pH_i_ upon acid exposure. We exposed *S. cerevisiae* wild-type to concentrations of HS and HA that inhibited growth by 50 and 100% in our previous work. For acetic acid this corresponded to 40 mM and 70 mM, while for HS 1 mM and 2 mM were used. Growth and pH_i_ profiles are shown in Figure 2. Addition of WOA caused immediate acidification (Figure 2B). With 40 mM of HA, recovery started instantaneously, and pH_i_ was restored almost to the pH_i_ of unstressed cell after 80 min (Figure 2B). However, no pH_i_ recovery was observed with 70 mM HA stress in the time frame studied, and growth was not resumed (Figures 2A,B). Cellular ATP was depleted by 60% in 40 mM HA stressed cells. Remarkably, 70 mM caused only a 21% reduction (Figure 2C). ADP was also reduced in both conditions, but again more at moderate than at severe stress (Figure 2D).

The HS concentrations used in these conditions did not recapitulate the 60 and 90% growth inhibition observed in our previous work (Orij et al., 2009; Ullah et al., 2012), possibly due to a different experimental setup. Growth inhibition and acidification were slightly higher with 2 mM than with 1 mM HS stress. We did not observe recovery of either pH_i_ or growth within the time frame (Figures 2E,F).

With 1 mM HS, ATP levels decreased by ~51% at *t* = 0.5 h and did not recover till *t* = 4 h. Decrease of ATP after 2 mM HS stress was less than after 1 mM HS (Figure 2G). The ADP concentrations were decreased by moderate stress conditions, while no significant decrease was observed at the higher dosage.

### Reduced ATP consumption by proton pumping does not improve growth

We hypothesized that if ATP depletion is a stronger cause of growth inhibition than the accumulation of protons or anions itself, decreasing the pumping activity might actually improve growth. Since *PMA1* is an essential gene, we used the *pma1* hypomorph *pma1-007* (Porat et al., 2005), which has only 50% Pma1p expression and activity, to assess the effect of reduction of proton pumping associated ATP consumption on the growth inhibitory effect of sorbic and acetic acid.

**Figure 3:**
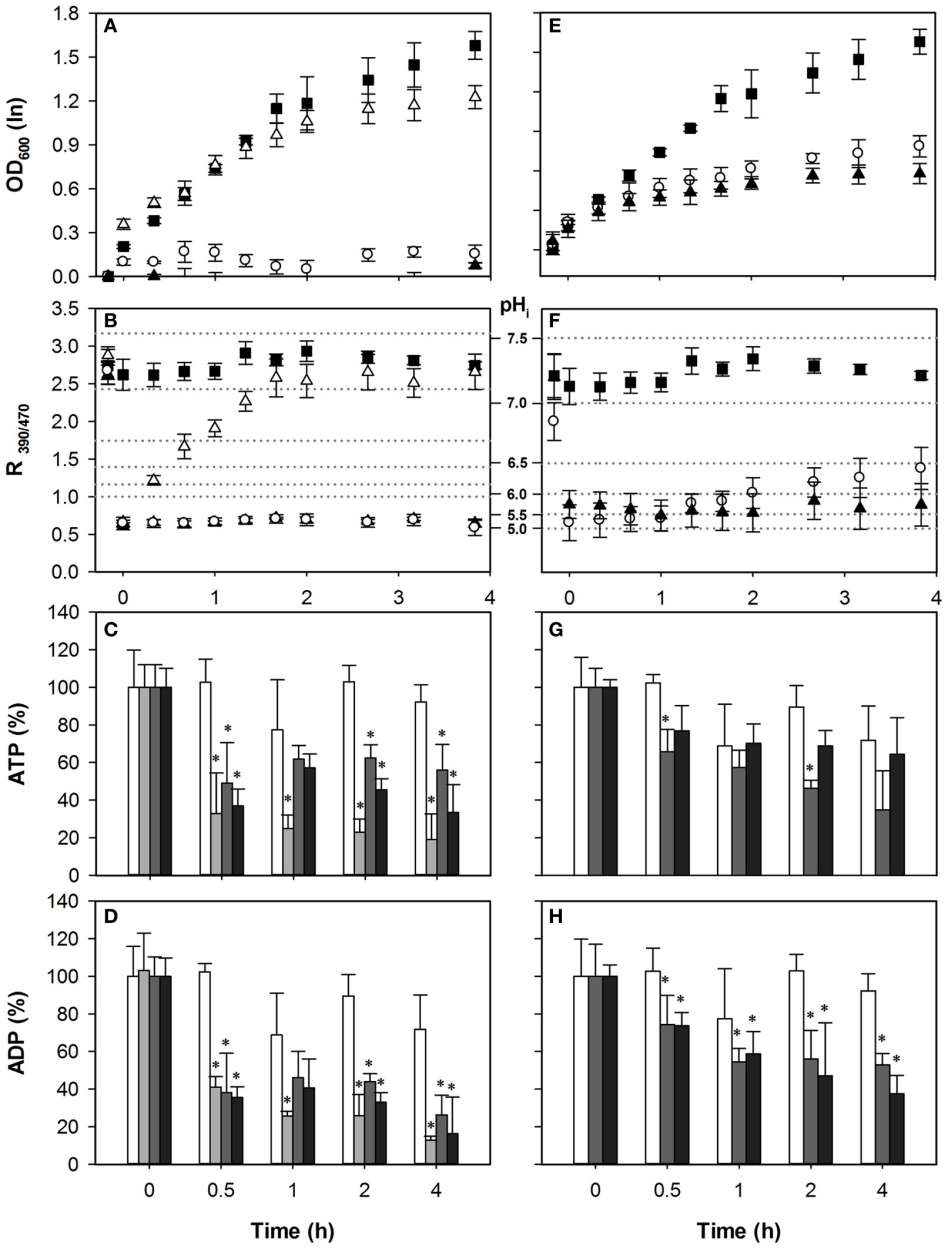
**Effect of acetic acid (left hand panels) and sorbic acid (right hand panels) on growth (A,E), intracellular pH (B,F), cellular ATP (C,G), and ADP (D,H) concentrations of *pma1-007***. Growing cultures were exposed to 0 mM (■ and white bars), 25 mM (△ and light gray bars), 40 mM HA or 1 mM HS (◦ and dark gray bars), and 70 mM HA or 2 mM HS (▲ and black bars) of HA/HS at *t* = 0. pH_i_ data are presented as ratio of fluorescence emission upon excitation at 390 nm and 470 nm (R_390/470_) while the relevant pH_i_ values derived from the calibration curves are also indicated. Data presented are the average of three independent cultivations and error bars represents standard deviation. An asterisk indicates significance of the difference between acid exposed and control cultures at similar time point (two-tailed *t*-test; *P* < 0.05). 40 mM HA or 1 mM HS.

Cells with reduced Pma1p activity did not show hypersensitivity to HS compared to wild-type (Figure 3E), and pH_i_ reduction and recovery were similar, consistent with our previous experiments (Ullah et al., 2012). Growth recovery of *pma1-007* coincided with pH_i_ restoration during HS stress. The ATP concentration initially decreased less than in wild-type, with only 28% for 1 mM, compared to 51% in wild-type. Upon 1 mM HS stress, it did however continue to decrease over the course of time. Two millimolar HS stress resulted in an ATP depletion similar to the wild-type (Figure 3G). In both stress conditions ADP levels were significantly reduced by the addition of the acid (Figure 3H). However, the reduced ATP consumption did not lead to an alleviation of the growth inhibition, suggesting that the proton extrusion coupled ATP consumption is less of a cause of growth inhibition than is the acidification itself.

We have shown previously that for HA, acidification correlates strongly with growth inhibition, and the effect of HA on growth and pH_i_ was enhanced by decreasing Pma1p activity (Ullah et al., 2012). Indeed, the mutant *pma1-007* was more sensitive to HA. Even moderate stress (40 mM) had a severe effect on growth of the mutant compared to wild-type (Figure 3). Therefore, to still assess the effect of a 50% growth inhibitory concentration, we included a 25 mM HA stress condition. Thus, three concentrations (25, 40, and 70 mM) of acetic acid stress were added to growing cultures of *pma1-007* cells. The pH_i_ of *pma1-007* dropped below pH 5.0 with all three concentrations, and growth and pH_i_ could be restored after the 25 mM HA stress only (Figures 3A,B). As observed with wild-type, the lower concentration of HA (25 mM) caused a strong depletion of ATP, while the higher concentrations (40 and 70 mM) actually led to a smaller reduction of ATP (Figure 3C).

Interestingly, growth of *pma1-007* was hardly affected by 25 mM HA stress, but the drop in the cellular ATP was prominent and ATP remained low up to 4 h after the initial stress (Figure 3C). ADP concentrations were also decreased compared to control (Figure 3D). Therefore, reduction of Pma1p activity did not result in higher ATP concentrations upon stress, and did not alleviate the stress phenotype. Rather, it enhanced the effect of acetic acid stress because of insufficient proton extrusion capacity. It appears, therefore, that the ATP consumption by the proton pump does not contribute strongly to the growth inhibitory effect of these two weak acids, increased acidification does.

### Anion expulsion contributes strongly to ATP consumption

**Figure 4:**
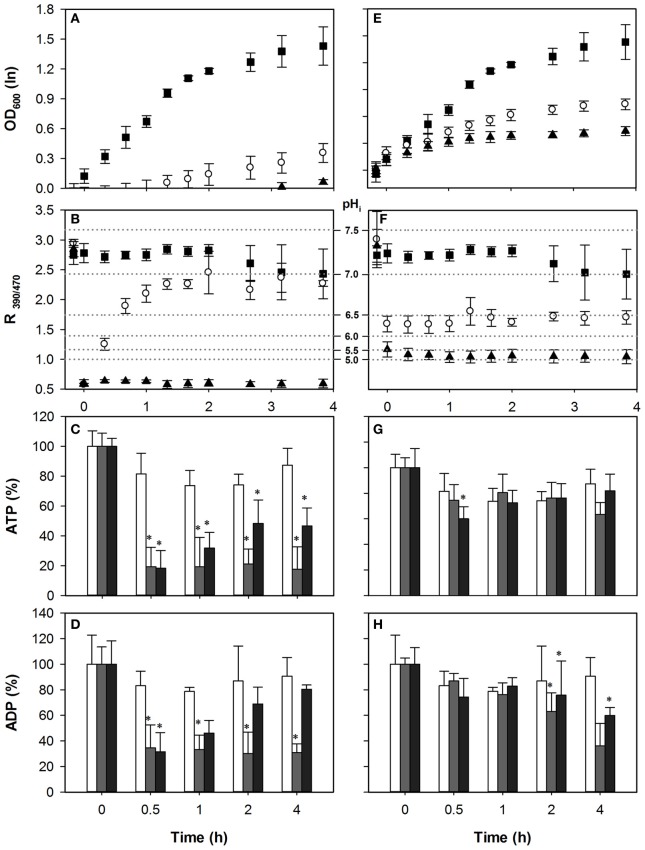
**Effect of acetic acid (left hand panels) and sorbic acid (right hand panels) on growth (A,E), intracellular pH (B,F), cellular ATP (C,G), and ADP (D,H) concentrations of *pdr12*Δ**. Growing cultures were exposed to 0 mM (■ and white bars), 40 mM HA or 1 mM HS (◦ and dark gray bars), and 70 mM HA or 2 mM HS (▲ and black bars) at *t* = 0. pH_i_ data are presented as ratio of fluorescence emission upon excitation at 390 nm and 470 nm (R_390/470_) while the relevant pH_i_ values derived from the calibration curves are also indicated. Data presented are the average of three independent cultivations and error bars represents standard deviation. An asterisk indicates significance of the difference between acid exposed and control cultures at similar time point (two-tailed *t*-test; *P* < 0.05).

The role of Pdr12p under acetic acid stress remains ill-understood. Pdr12p is not required for HA resistance and its deletion has no effect on growth in the presence of HA (Bauer et al., 2003; Ullah et al., 2012). Since Pdr12p expression is not induced by HA and its activity is not required for the extrusion of anions or protons during HA stress, we predicted that deletion of the gene would not affect adenylate nucleotide concentrations upon acetic acid stress, but would do so in case of HS exposure. Indeed, upon acetic acid stress, the growth profile of *pdr12*Δ was similar to the wild-type (Figure 4A). Both concentrations led to a pH_i_ decrease to below 5, and as with wild-type pH_i_ could be restored only in the 40 mM HA stress condition (Figure 4B). The pattern of adenylate nucleotides observed in *pdr12*Δ was similar to that of wild-type, (Figures 4C,D) corroborating the idea that Pdr12p activity does not contribute to ATP depletion in case of acetic acid stress. Cellular ADP concentrations were also reduced (Figure 4D) again more strongly at lower stress.

Pdr12p expression is induced by sorbate and benzoate after ~60–90 min of acid exposure to levels almost as high as Pma1p (Piper et al., 1998; Papadimitriou et al., 2007). The pH_i_ of *pdr12*Δ cultures decreased to 6.5 and 6.0 upon exposure to 1 mM and 2 mM of HS, respectively (Figure 4F). Upon exposure to 1 mM HS, *pdr12*Δ cells recovered growth after 4 h of adaptation (Figure 4E). However, cells treated with 2 mM HS did not recover growth and pH_i_, in line with our previous analysis. If Pdr12p activity was a major energy consuming factor upon acid stress, we would expect that deletion of *PDR12* would reduce ATP consumption compared to wild type in the case of HS stress. Indeed, in contrast to the wild type cells, ATP levels were decreased only little during sorbic acid stress from *t* = 0.5 to *t* = 4 h as shown in Figure 4G. The drop in the ATP was 7% in *pdr12*Δ cells with 1 mM of sorbic acid, and 21% with 2 mM, in which case the ATP contents were mostly recovered during the time course (Figure 4G). No significant difference was observed in cellular ADP concentrations during the first hour of stress exposure (Figure 4H).

## Discussion

WOAs are widely used food preservatives in food and beverage industries because they are particularly efficacious toward yeast and fungal spoilage (Winter et al., 2008). Characteristically, weak-acid preservatives do not kill yeast but rather inhibit growth (Lambert and Stratford, 1999), and this ability of yeasts to survive and proliferate in the presence of WOA is an important spoilage factor. Various mechanisms are thought to contribute to the growth inhibitory effect, but quantitative mechanistic understanding is still lacking.

We showed that the adaptation to sorbic or acetic acid resulted in a decreased diffusional entry of the molecule. It has been suggested previously that adapted cells decrease the diffusional entry of acids by remodeling their plasma membranes and reinforcement of cell wall structure to decrease its porosity (Mira et al., 2010a). Furthermore, acidification is directly affected by the extent to which the cell can buffer the protons that are released. A recent study assessed the buffering capacity of living cells, and showed this to be higher than previously estimated (Maresova et al., 2010). However, this analysis of buffer capacity determined not only the passive buffering, by proton association to intracellular weak acids, but a combination of this passive buffering with active extrusion of protons to counteract the diffusional entry (Mollapour et al., 2008). To distinguish between the passive contributions of altered membrane composition and/or intracellular buffer capacity, and active proton efflux, we analyzed how the initial acidification rate depended on both of these aspects. Because the rate of acidification was not dependent on the presence of an energy source it reflects acid entry. Therefore, we concluded that pre-exposed cells indeed decrease the entry by alteration of either the structure of the plasma membrane or their cell wall composition, or by increasing intracellular buffering. Additionally, pH *recovery* depended strongly on the presence of an energy source.

### Effect of WOA on the cellular concentration of ATP

The intracellular concentration of ATP depends on the balance between energy production and consumption. Acetic and sorbic acid are known to stimulate different response in yeast, but in both stress conditions *S. cerevisiae* uses energy dependent membrane transporters to expel protons and anions, which may deplete the cellular ATP pool and thus inhibit growth (Breeuwer et al., 1994; Holyoak et al., 1999; Mollapour et al., 2008). HS was previously shown not to affect ATP production (Holyoak et al., 1996). Therefore we assume that a decrease in ATP observed is due to increased consumption, likely because of the activities of Pma1p and Pdr12p, the major proton and anion exporter during HS stress in *S. cerevisiae* (Morsomme et al., 2000; Mollapour et al., 2008). We showed that cells do use ATP in the presence of WOA but its depletion is not a major contribution to growth inhibition in the case of acetic acid: If ATP depletion would be the major cause of growth inhibition, then reduction of ATP dependent extrusion of WOA protons and anions should increase cellular ATP, and this should then relieve the growth inhibitory effect of the WOA compared to the drop in pH. Decreased expression of Pma1p in the *pma1-007* mutant slightly reduced ATP consumption upon in response to HA exposure, but the mutant was more sensitive rather than less (Stratford et al., 2009; Ullah et al., 2012). This increased susceptibility of *pma1-007* is likely due to the increased acidification rather than ATP consumption. As expected, growth, pH_i_ and intracellular ATP levels in *pdr12*Δ cells were similar to the wild-type cells, reconfirming that Pdr12p is not instrumental in HA tolerance (Mollapour et al., 2008). ATP consumption for proton expulsion is not a major cause of growth inhibition in case of HS stress either: Reduction of ATP consumption by Pma1p indeed resulted in increased ATP concentrations, but this did not alleviate the growth inhibitory effect of the acid. Interestingly, deletion of *PDR12* also reduced ATP consumption upon sorbic acid exposure, and this now correlated with improved growth behavior [as observed before Ullah et al. (2012)]. This reconfirms that Pdr12p is active in response to HS (Holyoak et al., 1996, 1999; Piper et al., 1998), and suggests that its activity is indeed a significant ATP burden. Therefore, Pma1p and Pdr12p are active during HS stress, but neither the proton pumping nor the anion expulsion activities is truly key for adapted growth in the timeframe observed in the presence of these low dosages of sorbic acid in *S. cerevisiae* (Papadimitriou et al., 2007; Ullah et al., 2012). Because the growth inhibition phenotype of sorbic acid was slightly alleviated in the *pdr12*Δ mutant, we conclude that the ATP consumption by Pdr12p is a larger burden to cellular functioning than is the sorbate anion accumulation.

Overall, we observed that when cells were subjected to severe WOA stress, they reduced the ATP expenditure for the recovery of either growth or pH_i_. This suggests that yeast has evolved mechanisms that prevent WOA induced ATP depletion, since ATP levels were higher in severe stress conditions where growth was completely inhibited, compared to moderate stress where ATP levels were lower but growth was partially inhibited. This phenomenon was seen with both acetic acid, where we have shown previously that growth inhibition was mainly caused by acidification (Ullah et al., 2012), and with the more lipophilic sorbic acid, where we expected other aspects of WOA to lead to growth inhibition. This implies that cells somehow sense the severity of the stress and change the strategy of adaptation, not consuming ATP for futile attempts at recovery, but rather reserving it, in spite of the low pH_i_ and high anion concentrations that now necessarily persist in the cell. Indeed, the H^+^-ATPase was shown to be inhibited by a chaperone, Hsp30p, which is induced upon stress (Piper et al., 1997). This should then be advantageous for later recovery of growth. Interestingly, we have recently shown that yeast encounters similarly low pH_i_ values every growth cycle, since upon glucose depletion, the cytosolic pH eventually drops to environmental pH (Orij et al., 2012), without consequences for cell viability in the course of several days. Because a low pH_i_ is a (proxy of a) signal for nutrient depletion or pending energy limitation (Dechant et al., 2010; Young et al., 2010; Orij et al., 2012), it is tempting to speculate that this signal could be used for safeguarding proper energy homeostasis or distribution upon nutrient depletion, to ensure stationary phase survival (Thomsson et al., 2005). Indeed, addition of glucose causes rapid phosphorylation of Pma1p, leading to its activation (Lecchi et al., 2007). This fits the notions of the interaction of nutrient sensing and growth control in response to environmental stress, where optimization of efficiency was also shown to be of key importance (Vilaprinyo et al., 2010; Zakrzewska et al., 2011). Understanding how pH_i_ affects cellular decision making to control the biochemical activity could provide powerful tools in understanding the biological mechanism of stress response and adaptation.

### Conflict of interest statement

The authors declare that the research was conducted in the absence of any commercial or financial relationships that could be construed as a potential conflict of interest.
